# CDON gene contributes to pituitary stalk interruption syndrome associated with unilateral facial and abducens nerve palsy

**DOI:** 10.1007/s13353-021-00649-w

**Published:** 2021-07-08

**Authors:** Monika Obara-Moszyńska, Bartłomiej Budny, Małgorzata Kałużna, Katarzyna Zawadzka, Aleksander Jamsheer, Anna Rohde, Marek Ruchała, Katarzyna Ziemnicka, Marek Niedziela

**Affiliations:** 1 Department of Pediatric Endocrinology and Rheumatology, Poznan University of Medical Sciences, 27/33 Szpitalna Str, 60-572 Poznan, Poland; 2 Department of Endocrinology, Metabolism and Internal Medicine, Poznan University of Medical Sciences, 49 Przybyszewskiego Str., 60-355 Poznan, Poland; 3 MNM Diagnostics Sp. z o.o., 64 Macieja Rataja Str., 61-695 Poznan, Poland; 4 Department of Medical Genetics, Poznan University of Medical Sciences, 8 Rokietnicka Str, 60-806 Poznan, Poland

**Keywords:** *CDON* gene, PSIS, Pituitary insufficiency, Facial nerve palsy, Abducens nerve palsy

## Abstract

The relationship between congenital defects of the brain and facial anomalies was proven. The Hedgehog signaling pathway plays a fundamental role in normal craniofacial development in humans. Mutations in the sonic hedgehog (SHH) signaling gene *CDON* have been recently reported in patients with holoprosencephaly and with pituitary stalk interruption syndrome (PSIS). This study’s aim was an elucidation of an 18-year-old patient presenting PSIS, multiple pituitary hormone deficiency, and congenital unilateral facial and abducens nerve palsy. Additionally, bilateral sensorineural hearing loss, dominating at the right site, was diagnosed. From the second year of life, growth deceleration was observed, and from the age of eight, anterior pituitary hormone deficiencies were gradually confirmed and substituted. At the MRI, characteristic triad for PSIS (anterior pituitary hypoplasia, interrupted pituitary stalk and ectopic posterior lobe) was diagnosed. We performed a comprehensive genomic screening, including microarrays for structural rearrangements and whole-exome sequencing for a monogenic defect. A novel heterozygous missense variant in the *CDON* gene (c.1814G > T; p.Gly605Val) was identified. The variant was inherited from the mother, who, besides short stature, did not show any disease symptoms. The variant was absent in control databases and 100 healthy subjects originating from the same population. We report a novel variant in the *CDON* gene associated with PSIS and congenital cranial nerve palsy. The variant revealed autosomal dominant inheritance with incomplete penetrance in concordance with previous studies reporting *CDON* defects.

## Introduction

The relationship between congenital defects of the brain and facial anomalies was proven (Kjaer 1995; Roitman and Laron 1978). In patients who present facial dysmorphia, which coexists with pituitary hormone deficiency symptoms, abnormalities of the hypothalamic-pituitary area should be suspected. There are many known genes involved in anterior pituitary development like *HESX*, *LHX3*, *LHX4*, *POU1F1*, *PROP1*, *SIX6*, *OTX2*, *PTX2*, *GLI2*, *SOX2*, and *SOX3.* Variants in these genes are associated with pituitary dysfunction, including combined pituitary hormone deficiencies (CPHDs) and isolated growth hormone (GH) deficiency (Davis 2013; Kelberman et al. 2009). However, little is known about the genes involved in the development of the pituitary stalk and posterior pituitary. Pituitary stalk interruption syndrome (PSIS) is a disorder characterized by the combination of three abnormalities found on magnetic resonance imaging (MRI): interrupted pituitary stalk, absent or ectopic posterior pituitary, and anterior pituitary hypoplasia. Besides the pituitary insufficiency, PSIS can be associated with other midline and ophthalmic abnormalities (Bashamboo et al. 2017; Pinto et al. 1997). In most cases, etiology of PSIS is unknown. Recently, molecular defects in various genes have been associated with PSIS, albeit in a small number of cases (Brauner et al. 2020). These findings suggest that PSIS belongs to the spectrum of holoprosencephaly-related defects (Voutetakis et al. 2016). Several genes as *PIT1*, *PROP1*, *LHX3/LHX4*, *TGIF*, *OTX2*, *HESX1*, *SOX3*, *PROKR2*, *ROBO1*, *WDR11*, and *GPR161* have been postulated to be associated with PSIS (Bashamboo et al. 2017; Davis 2010; Karaca 2015; McCormack et al. 2017; Wang et al. 2017; Ziemnicka 2016). Mendelian forms of PSIS are detected infrequently (< 5%), and a polygenic etiology has also been suggested (Guo 2017; Zwaveling-Soonawala 2018). Recently, variants in holoprosencephaly (HPE)-related genes have been reported in two patients with PSIS or isolated pituitary hypoplasia, presenting with CPHD and without HPE (Tatsi 2013). CDON (cell adhesion associated oncogene regulated) is a cell surface sonic hedgehog (SHH)–binding protein that promotes SHH signaling activity by acting as a coreceptor with PTCH1 (Kang et al. 1998). The Hedgehog signaling pathway plays a fundamental role in orchestrating normal craniofacial development in vertebrates (Xavier et al. 2016). Recently, heterozygous, loss-of-function *CDON* variants have been reported in six HPE patients with different phenotypic severity (Bae et al. 2011). Other authors identified a novel heterozygous nonsense variant in the *CDON* gene in a case of PSIS without HPE (Bashamboo et al. 2016) or puberty disturbances (Brauner et al. 2021).

Here, we describe a novel heterozygous missense variant in the *CDON* gene associated with PSIS and unilateral facial and abducens nerve palsy.

## Materials and methods

The birth weight was compared to reference data established by Niklasson and Albertsson-Wikland (2008). The height standard deviation score (htSDS) for chronological age was calculated using Polish references (Kulaga 2010) Bone age was estimated according to Greulich and Pyle method (Greulich and Pyle 1959).

### Patient report

The girl was born prematurely by cesarean section at 34 weeks of gestation, due to placental abruption. Her birth weight was 2160 g (between the mean and − 1SD compared to the normal range for a gestational week), and the Apgar score was 7/8/9 at 1, 5, and 10 min respectively. Clinical signs of intrauterine infection were diagnosed. At birth, she suffered cornea damage in her right eye because of the congenital palsy of her right facial nerve and inability to close this eye. Her right eye was exophthalmic, rotated upwards, the total loss of corneal epithelium was observed, and ulceration of the cornea developed. Additionally, transient glaucoma of both eyes was observed. On the second day of life, hematotympanum of the right ear was recognized, and paracentesis was performed. Due to neurological estimation, congenital, peripheral facial, and abducens nerve palsy at the right site were diagnosed. The girl also presented a superficial cavernous hemangioma below her right scapula. With time right, vesicourethral reflux grade 3 (because of dilation of urinary system and recurrent infections surgery was performed later on at age eight years), and bilateral sensorineural hearing loss (severe in the right ear, a slight degree in the left ear) were recognized. Her hearing impairment is effectively aided with an audio prosthesis based on the Bicros system.

When she was 2 years old, she underwent a surgical intervention of decompression of her right facial nerve, which consisted of a widening of the facial nerve canal. However, it was not successful. At the age of 4, she was examined in the Ophthalmology Department in Amiens, where Moebius syndrome was suggested, which consists usually of congenital, bilateral facial, and abducens nerve paralysis. The permanent strabismus of the right eye with an angle range of approximately 45 was observed. It was suggested that the strabismus could be operated on, but the surgery would not restore abduction. Amblyopia in the patient’s right eye due to vascular opacity of the lower part of the cornea was also suspected. Based on this opinion, the girl was later on operated. The gold plate was implanted into the right upper eyelid, and the temporal muscle was transplanted to the area of the mouth to improve the tension of this region. Therefore, she can close her right eye almost completely, yet her right lateral rectus muscle remained paralyzed, and she is unable to move her right eye horizontally. Still, the right corner of the mouth is lower.

**Fig. 1:**
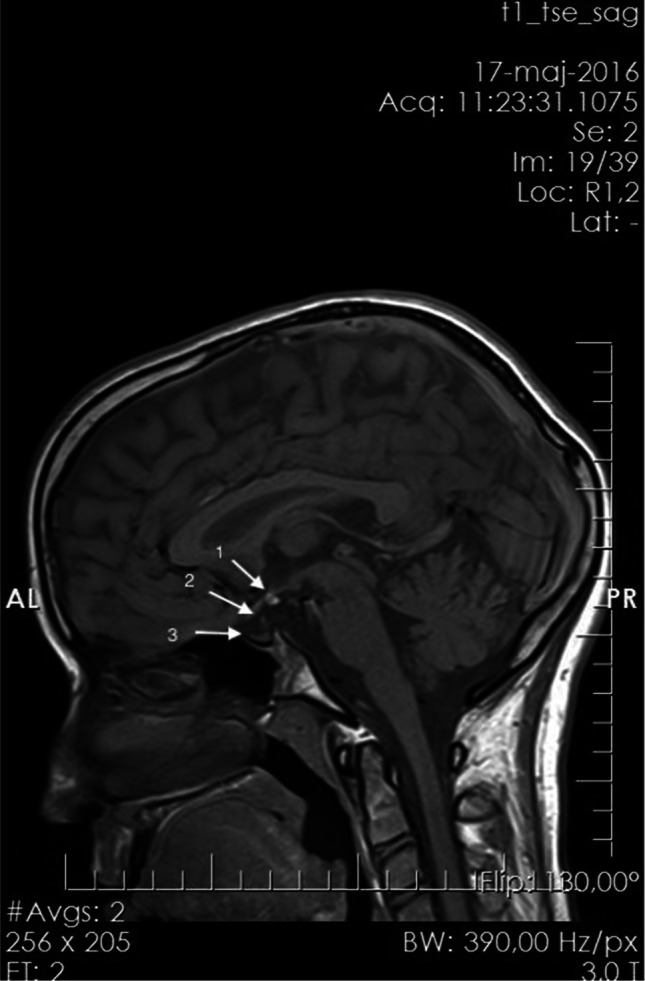
MRI image (sagittal plane) of the head of the presented patient showing typical triad of PSIS: ectopic posterior lobe (1), agenesis of the pituitary stalk (2), and hypoplastic anterior lobe (3). This examination came when the girl was 17 years old

**Fig. 2:**
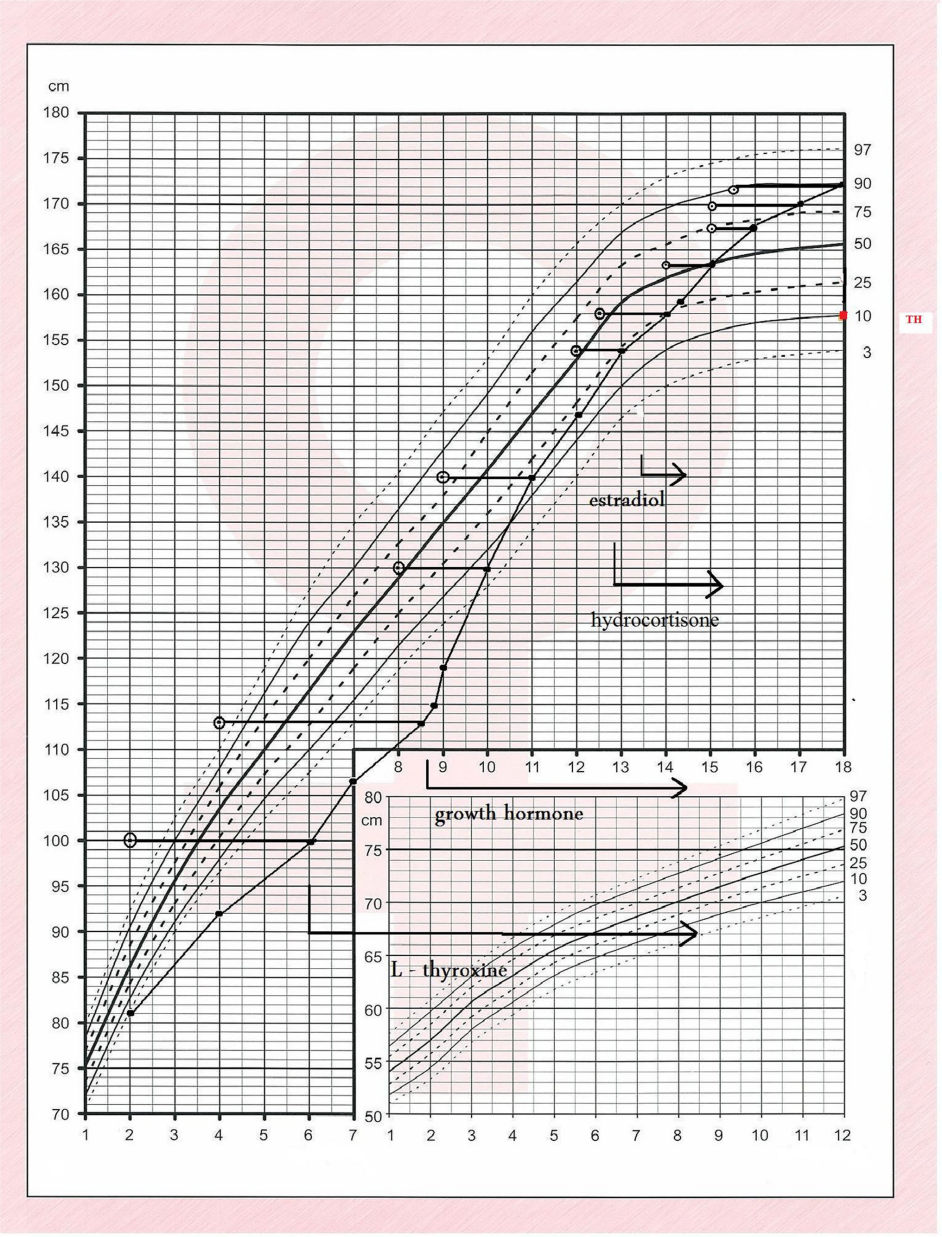
Patient’s growth chart

When she was 2 years old, her growth ratio started to decline. Her target height based on parental heights was 158 cm (10th centile, mother 154 cm, father 175 cm). At the age of six, the girl came to the Department of Pediatric Endocrinology and Diabetes, Poznan University of Medical Sciences. She was diagnosed with secondary thyroid insufficiency, and supplementation with l-thyroxine was ordered. At the age of 8 years, a complete GH deficiency was diagnosed. In all tests (during sleep and two pharmacological tests: insulin and clonidine), the maximal GH release was extremely low (0.2 ng/ml). IGF-1 level was also decreased (70 ng/ml; ref. value 175–445 ng/ml) (Table 1). MRI showed hypoplasia of the anterior lobe of the pituitary (height < 2 mm), interrupted pituitary stalk, and ectopic posterior lobe located within hypothalamus—indicating PSIS (Fig. 1). She started treatment with recombinant GH (rGH; Omnitrope, Sandoz) at the age of 8 years and 8 months. Her height at this point was 115 cm (− 3.0 SD). Her bone age was delayed by 4 years compared to her chronological age. rGH therapy resulted in a highly satisfactory effect (Fig. 2). At the age of 13 years, due to the lack of pubertal features and very low gonadotropin concentrations in response to LHRH stimulation, hypogonadotropic hypogonadism was diagnosed. Although the patient did not present characteristic symptoms for adrenal insufficiency at this same age, the ACTH test was performed, which revealed decreased levels of cortisol (Table 1). The substitution therapy with hydrocortisone and estradiol was started. In several measurements, hyperprolactinemia was confirmed. Macroprolactinemia was excluded.

**Table 1:** Laboratory results indicating combined pituitary insufficiency and hyperprolactinemia

Laboratory test	Result	Age in which the test was done [years]	Reference value
Max GH after onset of sleep [ng/ml]	0.2	8	> 10
Max GH after clonidine [ng/ml]	0.2	8	> 10
Max GH after insulin [ng/ml]	0.2	8	> 10
Max GH after glucagon [ng/ml]	0.2	18.5	> 10
IGF-1 [ng/ml]	70	8	175–445
IGFBP3 [ng/ml]	1279	8	3156–4839
TSH (IU/l]	0.039	11	0.35–4.94
ACTH [pg/ml]	12.5	13	10–60
Cortisol after ACTH (1 ug/1.73 m2) [ng/ml]	8.0	13	> 180
Cortisol after glucagon [ng/ml]	< 10	18.5	> 180
Estradiol [pg/ml]	< 20	13	Depending on age and cycle phase
LH after LHRH [mIU/ml]	< 0.5	13	Depending on age and cycle phase
FSH after LHRH [mIU/ml]	< 0.37	13	Depending on age and cycle phase
DHEA-S [umol/l]	0.07	13	1.02–7.16
PRL [ng/ml]	66.87	15	5.18–26.53

At present, the girl is 18.5 years old, and her height is 172 cm (+ 1.1 SD) and weight 58.2 kg. Her pubertal status is as follows: Ax 4, Pub 4, Th 4 on Tanner scale. Bleeding from the uterus on hormonal therapy appeared at the age of 17 years. She is being treated with l-thyroxin, rGH (metabolic dose), hydrocortisone, and combined therapy of 17-beta-estradiol and synthetic progestogen—dydrogesterone. Her psychological development is normal, and she will undertake biotechnology studies at the university.

**Fig. 3:**
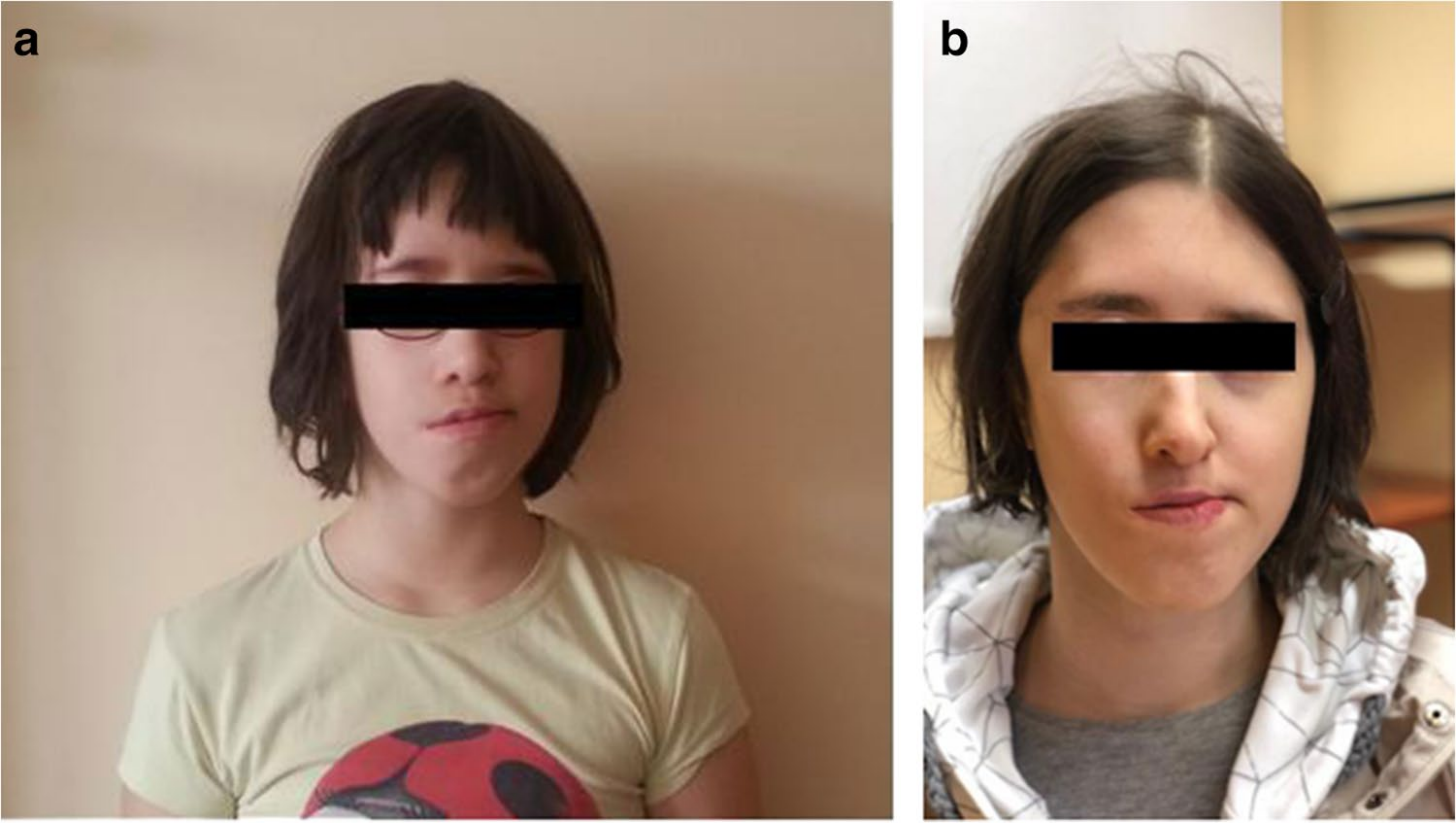
The patient’s facial appearance at the age of 13 (**a**) and 18 (**b**) years

The patient’s facial appearance at the age of 13 and 18 is shown in Fig. 3A and B. Further plastic surgery to her face is planned.

### Clinical characteristics of proband’s mother, family data

The patient’s mother was admitted to the Department of Endocrinology at age 55. She presented with short stature (height 152 cm), primary hypothyroidism caused by autoimmune thyroiditis, diabetes mellitus type 2, hypertension, obesity, and right renal artery duplication. At the moment of admission, she had a normal pituitary function. Ophthalmological consultation confirmed normal eye bulbs position with symmetrical movements and pupils’ reaction to light. Currently, she is a far-sighted person who needs glasses with moderate correction. She did not give consent for pituitary MRI. Patient’s father did not show any disorder or height disturbances, although he refused thorough clinical examination. Both parents gave consent to perform genetic analysis of CDON gene in trio. Clinical data of other family members are not available.

### Genetic testing

All analyses were conducted on DNA extracted from peripheral blood, using the phenol–chloroform method. A high-resolution microarray scanning for genomic imbalances was conducted with the use of Affymetrix CytoScanHD arrays (2,7 M, Santa Clara, USA) and GeneChip Scanner 3000 7G (Budny et al. 2020a). For an assay, 200 ng of genomic DNA was used. Array scans were managed using the Affymetrix Chromosome Analysis Suite (ChAS). Log2 R ratios and B-allele frequencies were used for copy number variations (CNVs) identification in the sample. To search for variants in known genes involved in pituitary organogenesis, patients were routinely screened using a targeted NGS panel for point variants in CPHD genes, as reported previously (Budny 2020b) with the use of ION Torrent platform (Thermo Scientific, USA). For whole-exome sequencing (WES), a 1 μg of genomic DNA from the subject was used for the construction of a library with the TruSeq DNA Sample Preparation and Enrichment Kit (Illumina). The achieved minimal mean depth of target regions was 230 × (post-alignment), and an average read length of 145 bp. 99% of the reads was aligned and mapped to the human genome reference sequence hg19 (BWA v0.7.12, Picard v1.130, GATK v3.4.0, SnpEff v4.1 g) of which 90.5% of non-redundancy. For pathogenicity evaluation, we used gene/transcript annotations (downloaded from UCSC GenomeBrowser, hg19), sequence variants from dbSNP (version 142), 1000 Genomes project (The 1000 Genomes Project Consortium, Phase3), ExAc Database (http://exac.broadinstitute.org/), and GenomAD v2.1.1(Lek 2016). Selected alterations, which were found using NGS, were confirmed using Sanger sequencing, and no discrepancy between these results was detected. The impact on protein structure and functionality was evaluated using following algorithms: Sift (Kumar et al. 2009), and PolyPhen2 (Adzhubei 2010), dbNSFP (Liu et al. 2016), FATHMM (Shihab 2013), MutationTaster v2 (Schwarz et al. 2014), PhenIX (Zemojtel 2014), CADD (Rentzsch et al. 2019), and HPO database (Kohler 2017). The variant prioritization was based on the variant frequency (novel or very rare variants), functional prediction using selected algorithms (VEP tool, Ensembl), and the possible contribution of genes into patient phenotype (HPO) (Jalali Sefid Dashti and Gamieldien 2017). A selected variant was classified according to ACMG recommendations (Richards 2015) with the use of the InterVar tool (Li and Wang 2017) and Varsome (Kopanos et al. 2019).

## Results

**Fig. 4:**
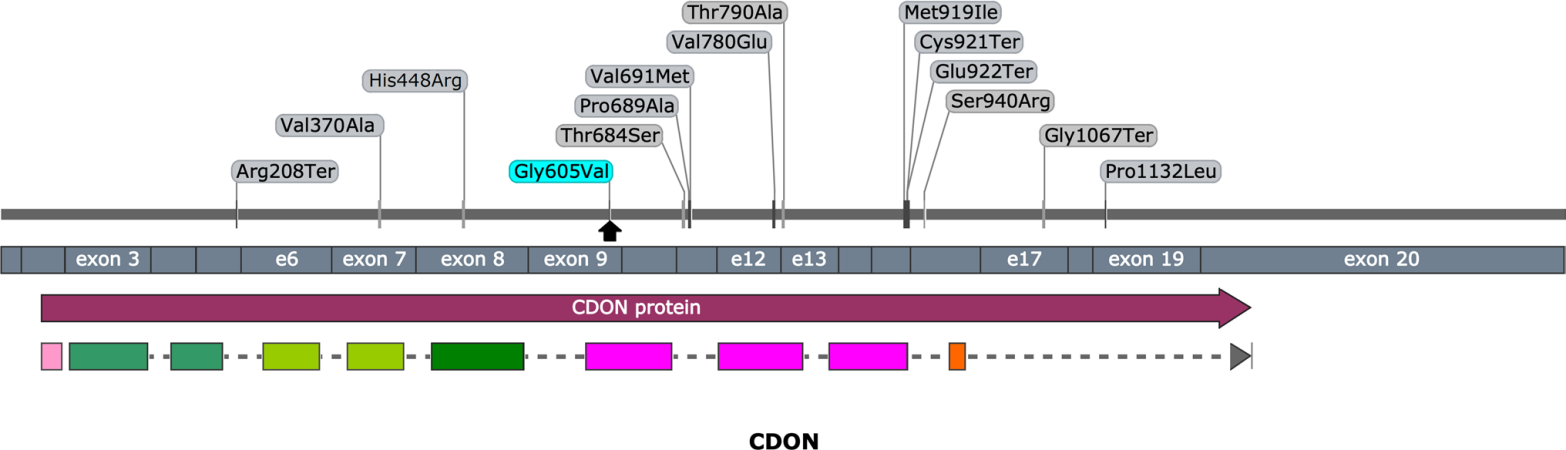
An overview of reported pathogenic variants in the *CDON* gene, according to the HGMD database. Variants are ordered in regard of nucleotide coding position (top section), exonic location (middle section), and protein sequence with highlighted functional domains (bottom section). Variants identified in this study were depicted with an arrow. The picture was prepared with the use of SnapGene software (GSL Biotech, snapgene.com)

The phenotype complexity and the proband’s appearance led to primary suspicion of Moebius syndrome (MBS). This suspicion prompted us to perform microarray testing and check abnormalities within 13q12 and 1p22, commonly contributing to the syndrome. No abnormalities were detected for the mentioned regions as well as for the other chromosomal locations. Having these data, we performed an NGS targeted analysis of genes contributing to CPHD and PSIS with no success of finding causative change. The eventual diagnosis was set after the WES testing, and a heterozygous missense variant in the *CDON* gene (c.1814G > T; p.Gly605Val, NM_001243597.1) was identified. This variant was predicted to be pathogenic in all used algorithms and was neither found in population databases. According to the ACMG criteria, the variant was classified as “Uncertain Significance” (PM2, PP3, PP4, BP1). We also checked the origin of this variant, and therefore a mother who is presenting subtle symptoms of the phenotype was tested using conventional bidirectional sequencing. This analysis showed that the mother is a carrier of this variant, presenting autosomal dominant transmission with incomplete penetrance. The variant position along the *CDON* gene and previously reported variants is shown in Fig. 4.

## Discussion

Pituitary development is a sophisticated and complex process, which leads to forming functionally different lobes. PSIS is defined as a classic triad of congenital defects described at MRI. Over the last years, the definition of PSIS has changed, and also patients with one abnormality such as ectopic posterior lobe or interrupted stalk are diagnosed with PSIS (Vergier et al. 2019). Our patient presents all the typical abnormalities described in this syndrome (ectopic posterior lobe, hypoplasia of anterior lobe, and interrupted pituitary stalk). Recent findings suggest that PSIS belongs to the spectrum of midline defects and is a mild form of HPE (Voutetakis et al. 2016). Discussing PSIS as a part of HPE, it must be pointed out that holoprosencephaly is the most common structural anomaly of human brain development with a prevalence of 1 in 250 conceptuses and 1 in 16,000 at birth (Yamada et al. 2004). HPE is associated with different brain development abnormalities. In the most severe form, alobar HPE is characterized by a single brain ventricle. Less severe forms are semilobar and lobar HPE (Zhang et al. 2006). The range of midline defects extends from most to least severe, referred to as the HPE spectrum (Ming and Muenke 2002). Associated craniofacial anomalies are also of different variety as cyclopia or more delicate like solitary median maxillary central incisor (Zhang et al. 2006). Neuroradiological investigations showed that PSIS per se could be associated with various extracerebral midline defects, which are responsible for different clinical manifestations. The midline defects involving the central nervous system are mainly consisting of brain and eye defects (Kulkarni et al. 2012).The ophthalmologic pathologies may include optic nerve hypoplasia and coloboma of the retina (Vergier et al. 2019). Some dysmorphic features of the face, such as hypotelorism, broad forehead with frontal bossing, high nasal bridge, and short chin, were also described in PSIS (El Chehadeh-Djebbar 2011; Karaca et al. 2015). From other abnormalities, there are coexisting defects of the skin, heart, skeleton, gastrointestinal, and urinary tract (Simon et al. 2006).

The *CDON* variants were described as the etiology factor of both HPE and PSIS (Bae et al. 2011; Bashamboo et al. 2016). However, only very few PSIS patients were diagnosed with one single molecular defect and Mendelian inheritance (Bar 2015). The clinical picture of PSIS can be variable, and the symptoms of the endocrine profile, as well as MRI abnormalities, vary within family members, frequently with mildly affected or unaffected variant-positive relatives (Simon et al. 2006; Vergier et al. 2019). About one-third of variants carriers in pedigrees do not exhibit a typical clinical phenotype of HPE, and it means that variants found in many patients with HPE are inherited from unaffected parents (Mercier 2011; Ming and Muenke 2002). These observations led to the hypothesis that HPE is caused by variants in which the penetrance and expressivity are enhanced by a second variant or the presence of cooperating modifier genes (Hong and Krauss 2012). Probably HPE is a multiple-hit disorder, which is an effect of a complex interplay of developmental, genetic, and environmental factors (Krauss 2007; Roessler and Muenke 2010; Roessler et al. 2012; Solomon, 2010). Hong et al. developed a mouse HPE model in which there is a synergy between a variant in the *CDON* gene and in utero exposure to ethanol, a suspected but unproven HPE risk factor. Loss of CDON and in utero ethanol exposure in 129S6 mice gave little or no phenotype individually, but together produced defects in early midline patterning, inhibition of SHH signaling in the developing forebrain, and a broad spectrum of HPE phenotypes (Hong and Krauss 2012).

The identified p.Gly605Val variant was also present in probands mother, who showed short stature (152 cm) associated with diabetes mellitus, metabolic syndrome, autoimmune thyroid disease (Hashimoto thyroiditis), and right renal artery duplication. No other clinical abnormalities were diagnosed, also regarding hormone level concentrations: cortisol, ACTH, GH, IGF-1, and PRL. The gonadotropins were at menopause levels. No toxic factors were referred to during pregnancy with presented patients, but the patients’ compliance is always a doubt.

*CDON* variants are commonly associated with different extracerebral abnormalities. Bae et al. described a broad HPE phenotype in patients showing *CDON* variants. Besides central nervous system abnormalities, they presented hepatic cholestasis, biliary atresia, or dark, thick eyebrows with synophrys (Bae et al. 2011).

Our patient with *CDON* variant besides PSIS presents additional neurological abnormalities—congenital nerve palsy of two cranial nerves—facial and abducens nerves. The girl also had right vesicourethral reflux and sensorineural hearing loss. The etiology of the hearing problems is unclear. It is possible that vestibulocochlear nerve (the next cranial nerve) was affected leading to the impairment of sensorineural hearing. The other etiology may be a consequence of antibiotic therapy given in the neonate period (vancomycin, netilmicin) or severe growth hormone deficiency. Muus et al. showed that hearing loss is prevalent in children with growth hormone deficiency GHD with a predisposition to be bilateral. The authors evidenced that insulin-like growth factor 1 (IGF-1) is important for inner-ear development (Muus et al. 2017).

The presented case is similar to that reported by Bashamboo et al. (2016). Incomplete penetrance of the PSIS phenotype resulted from nonsense variant p.Glu922Ter inherited from probands’ mother was noted. The mother showed normal height, no symptoms of hormonal disturbances, but she suffered for congenital convergent strabismus. Our patient also has unilateral strabismus due to the abducens nerve palsy. These findings would suggest possible CDON contribution to the development of nerves responsible for eye movement. PSIS manifests typically with anterior pituitary insufficiency. It can occur at birth with severe hormonal phenotype or later, during pediatric life appearing gradually (Vergier et al. 2019). Our patient presents multi-hormonal pituitary insufficiency, which developed gradually with TSH deficiency as the first, diagnosed at 6 years. The hormonal disturbances became the dominating health problem with the time. The elevated prolactin concentration is an effect of stalk agenesis and a lack of dopaminergic pathways.

In conclusion, we present novel evidence of *CDON* gene contribution to development, supporting previous reports of phenotypical complexity and incomplete dominant transmission.

## Data Availability

The authors confirm that the data supporting the findings of this study are available within the article or and on request at corresponding author.

## References

[CR1] Adzhubei IA (2010). A method and server for predicting damaging missense mutations. Nat Methods.

[CR2] Bae GU, Domene S, Roessler E, Schachter K, Kang JS, Muenke M, Krauss RS (2011). Mutations in CDON, encoding a hedgehog receptor, result in holoprosencephaly and defective interactions with other hedgehog receptors. Am J Hum Genet.

[CR3] Bar C (2015). Pituitary stalk interruption syndrome from infancy to adulthood: clinical, hormonal, and radiological assessment according to the initial presentation. PLoS One.

[CR4] Bashamboo A, Bignon-Topalovic J, Rouba H, McElreavey K, Brauner R (2016). A Nonsense Mutation in the Hedgehog Receptor CDON Associated with Pituitary Stalk Interruption Syndrome. J Clin Endocrinol Metab.

[CR5] Bashamboo A, Bignon-Topalovic J, Moussi N, McElreavey K, Brauner R (2017). Mutations in the human ROBO1 gene in pituitary stalk interruption syndrome. J Clin Endocrinol Metab.

[CR6] Brauner R, Bignon-Topalovic J, Bashamboo A, McElreavey K (2020). Pituitary stalk interruption syndrome is characterized by genetic heterogeneity. PLoS One.

[CR7] Brauner R, Bignon-Topalovic J, Bashamboo A, McElreavey K (2021). Peripheral Precocious puberty of ovarian origin in a series of 18 girls: exome study finds variants in genes responsible for hypogonadotropic hypogonadism. Front Pediatrics.

[CR8] Budny B, Karmelita-Katulska K, Stajgis M, Zemojtel T, Ruchala M, Ziemnicka K (2020a) Copy Number variants contributing to combined pituitary hormone deficiency. Int J Mol Sci 21. 10.3390/ijms21165757 10.3390/ijms21165757 PMC7461210 32796691

[CR9] Budny B (2020). SEMA3A and IGSF10 are novel contributors to combined pituitary hormone deficiency (CPHD). Front Endocrinol (lausanne).

[CR10] Davis SW (2010). Molecular mechanisms of pituitary organogenesis: in search of novel regulatory genes. Mol Cell Endocrinol.

[CR11] Davis SW (2013). Pituitary gland development and disease: from stem cell to hormone production. Curr Top Dev Biol.

[CR12] El Chehadeh-Djebbar S (2011). 17q21.31 microdeletion in a patient with pituitary stalk interruption syndrome. Eur J Med Genet.

[CR13] Greulich WW, Pyle SI (1959). Radiographic atlas of skeletal development of the hand and wrist.

[CR14] Guo QH (2017). Multi-genic pattern found in rare type of hypopituitarism: a whole-exome sequencing study of Han Chinese with pituitary stalk interruption syndrome. J Cell Mol Med.

[CR15] Hong M, Krauss RS (2012). Cdon mutation and fetal ethanol exposure synergize to produce midline signaling defects and holoprosencephaly spectrum disorders in mice. PLoS Genet.

[CR16] JalaliSefidDashti M, Gamieldien J (2017). A practical guide to filtering and prioritizing genetic variants. BioTechniques.

[CR17] Kang JS, Mulieri PJ, Miller C, Sassoon DA, Krauss RS (1998). CDO, a robo-related cell surface protein that mediates myogenic differentiation. J Cell Biol.

[CR18] Karaca E (2015). Whole-exome sequencing identifies homozygous GPR161 mutation in a family with pituitary stalk interruption syndrome. J Clin Endocrinol Metab.

[CR19] Kelberman D, Rizzoti K, Lovell-Badge R, Robinson IC, Dattani MT (2009). Genetic regulation of pituitary gland development in human and mouse. Endocrine Rev.

[CR20] Kjaer I (1995). Human prenatal craniofacial development related to brain development under normal and pathologic conditions. Acta Odontol Scand.

[CR21] Kohler S (2017). The Human Phenotype Ontology in. Nucleic Acids Res.

[CR22] Kopanos C, Tsiolkas V, Kouris A, Chapple CE, Albarca Aguilera M, Meyer R, Massouras A (2019). VarSome: the human genomic variant search engine. Bioinformatics.

[CR23] Krauss RS (2007). Holoprosencephaly: new models, new insights. Expert Rev Mol Med.

[CR24] Kulaga Z (2010). The height-, weight-, and BMI-for-age of Polish school-aged children and adolescents relative to international and local growth references. BMC Public Health.

[CR25] Kulkarni C, Moorthy S, Pullara SK, Rajeshkannan R, Unnikrishnan AG (2012). Pituitary Stalk Transection Syndrome: Comparison of Clinico-Radiological Features in Adults and Children with Review of Literature. Indian J Radiol Imaging.

[CR26] Kumar P, Henikoff S, Ng PC (2009). Predicting the effects of coding non-synonymous variants on protein function using the SIFT algorithm. Nat Protoc.

[CR27] Lek M (2016). Analysis of protein-coding genetic variation in 60,706 humans. Nature.

[CR28] Li Q, Wang K (2017). InterVar: Clinical interpretation of genetic variants by the ACMG-AMP Guidelines. Am J Hum Genet.

[CR29] Liu X, Wu C, Li C, Boerwinkle E (2016). dbNSFP v3.0: a one-stop database of functional predictions and annotations for human nonsynonymous and splice-site SNVs. Human Mutation.

[CR30] McCormack SE, Li D, Kim YJ, Lee JY, Kim SH, Rapaport R, Levine MA (2017). Digenic inheritance of PROKR2 and WDR11 Mutations in pituitary stalk interruption syndrome. J Clin Endocrinol Metab.

[CR31] Mercier S (2011). New findings for phenotype-genotype correlations in a large European series of holoprosencephaly cases. J Med Genet.

[CR32] Ming JE, Muenke M (2002). Multiple hits during early embryonic development: digenic diseases and holoprosencephaly. Am J Hum Genet.

[CR33] Muus JS, Weir FW, Kreicher KL, Bowlby DA, Discolo CM, Meyer TA (2017). Hearing loss in children with growth hormone deficiency. Int J Pediatr Otorhinolaryngol.

[CR34] Niklasson A, Albertsson-Wikland K (2008). Continuous growth reference from 24th week of gestation to 24 months by gender. BMC Pediatrics.

[CR35] Pinto G, Netchine I, Sobrier ML, Brunelle F, Souberbielle JC, Brauner R (1997). Pituitary stalk interruption syndrome: a clinical-biological-genetic assessment of its pathogenesis. J Clin Endocrinol Metab.

[CR36] Rentzsch P, Witten D, Cooper GM, Shendure J, Kircher M (2019). CADD: predicting the deleteriousness of variants throughout the human genome. Nucleic Acids Research.

[CR37] Richards S (2015). Standards and guidelines for the interpretation of sequence variants: a joint consensus recommendation of the American College of Medical Genetics and Genomics and the Association for Molecular Pathology. Genet Med.

[CR38] Roessler E, Muenke M (2010). The molecular genetics of holoprosencephaly American journal of medical genetics Part C. Seminars Med Genet.

[CR39] Roessler E, Velez JI, Zhou N, Muenke M (2012). Utilizing prospective sequence analysis of SHH, ZIC2, SIX3 and TGIF in holoprosencephaly probands to describe the parameters limiting the observed frequency of mutant genexgene interactions. Mol Genet Metab.

[CR40] Roitman A, Laron Z (1978). Hypothalamo-pituitary hormone insufficiency associated with cleft lip and palate. Arch Dis Childhood.

[CR41] Schwarz JM, Cooper DN, Schuelke M, Seelow D (2014). MutationTaster2: mutation prediction for the deep-sequencing age. Nat Methods.

[CR42] Shihab HA (2013). Predicting the functional, molecular, and phenotypic consequences of amino acid substitutions using hidden Markov models. Human Mutation.

[CR43] Simon D, Hadjiathanasiou C, Garel C, Czernichow P, Leger J (2006). Phenotypic variability in children with growth hormone deficiency associated with posterior pituitary ectopia. Clin Endocrinol (oxf).

[CR44] Solomon BD (2010). Analysis of genotype-phenotype correlations in human holoprosencephaly American journal of medical genetics Part C. Seminars Med Genet.

[CR45] Tatsi C (2013). Pituitary stalk interruption syndrome and isolated pituitary hypoplasia may be caused by mutations in holoprosencephaly-related genes. J Clin Endocrinol Metab.

[CR46] Vergier J, Castinetti F, Saveanu A, Girard N, Brue T, Reynaud R (2019). DIAGNOSIS OF ENDOCRINE DISEASE: pituitary stalk interruption syndrome: etiology and clinical manifestations. Eur J Endocrinol.

[CR47] Voutetakis A, Sertedaki A, Dacou-Voutetakis C (2016). Pituitary stalk interruption syndrome: cause, clinical manifestations, diagnosis, and management. Curr Opin Pediatrics.

[CR48] Wang CZ, Guo LL, Han BY, Su X, Guo QH, Mu YM (2017) Pituitary Stalk interruption syndrome: from clinical findings to pathogenesis. J Neuroendocrinol 29. 10.1111/jne.12451 10.1111/jne.12451 27917547

[CR49] Xavier GM, Seppala M, Barrell W, Birjandi AA, Geoghegan F, Cobourne MT (2016). Hedgehog receptor function during craniofacial development. Dev Biol.

[CR50] Yamada S, Uwabe C, Fujii S, Shiota K (2004). Phenotypic variability in human embryonic holoprosencephaly in the Kyoto Collection Birth defects research Part A. Clin Mol Teratol.

[CR51] Zemojtel T (2014). Effective diagnosis of genetic disease by computational phenotype analysis of the disease-associated genome. Sci Transl Med.

[CR52] Zhang W, Kang JS, Cole F, Yi MJ, Krauss RS (2006). Cdo functions at multiple points in the Sonic Hedgehog pathway, and Cdo-deficient mice accurately model human holoprosencephaly. Dev Cell.

[CR53] Ziemnicka K (2016). Two coexisting heterozygous frameshift mutations in PROP1 are responsible for a different phenotype of combined pituitary hormone deficiency. J Appl Genet.

[CR54] Zwaveling-Soonawala N (2018). Clues for polygenic inheritance of pituitary stalk interruption syndrome from exome sequencing in 20 patients. J Clin Endocrinol Metab.

